# Long-Term Ecosystem Monitoring Along the Trabocchi Coast (Chieti, Italy): Insights from Underwater Visual Surveys (2011–2024)

**DOI:** 10.3390/ani14233469

**Published:** 2024-12-01

**Authors:** Alessio Arbuatti, Alessandra Di Serafino, Pia Lucidi

**Affiliations:** 1 Department of Veterinary Medicine, University of Teramo, 64100 Teramo, Italy; aarbuatti@unite.it; 2 Center of Advanced Studies and Technology, University of Chieti, 66100 Chieti, Italy; alessandra.diserafino@unich.it; 3 Department of BioSciences and Technology for Food, Agriculture, and Environment, University of Teramo, 64100 Teramo, Italy

**Keywords:** Costa dei Trabocchi, ecosystem vulnerability, biodiversity, GSA17, habitat directive

## Abstract

This study explores the use of underwater visual surveys (UVSs) to assess biodiversity along the Trabocchi Coast of the Adriatic Sea, one of the few remaining areas with natural reefs in Italy’s middle Adriatic region GSA17. Fourteen years of observations underscore the effectiveness of UVSs in ecological monitoring and enhancing understanding of the Trabocchi reef’s biodiversity, which has thus far been minimally studied. The marine environment supports a complete and balanced trophic structure, from producers and invertebrates to vertebrate species, including herbivorous fish, a variety of predators, and even bottlenose dolphins. The Trabocchi Coast also serves as a nursery for commercially valuable species (56.5%) and hosts several “climate” indicator fish species (33.3%). Species of regulatory concern, such as *Lithophaga lithophaga* and *Cladocora caespitosa,* both listed under CITES Appendix II, as well as invasive species like *Rapana venosa* and the harmful dinoflagellate *Ostreopsis ovata,* which poses public health risks, are also found here. This work aligns with the 2023 National Biodiversity Strategy, emphasizing UVSs’ role in raising awareness and supporting the conservation of marine resources under increasing anthropogenic pressures. Sustainable coastal management and responsible tourism are particularly vital for preserving the fragile ecosystems along the Trabocchi Coast.

## Simple Summary

Underwater visual surveys (UVSs) offer valuable insights into both common and elusive species within reef environments, shedding light on how they respond to environmental changes. Despite Italy’s extensive 8300 km coastline, natural rocky stretches are rare along the Adriatic Sea, with one prominent example being Abruzzo’s Trabocchi Coast (Chieti, Italy), famously celebrated by the poet D’Annunzio. Fourteen years of research on this rocky habitat has enabled consistent monitoring of this vulnerable area, already at risk from sewage mismanagement, as well as potential flooding and landslide hazards. Beyond the economically significant species it supports, the shallow waters of the Trabocchi reef serve as habitats for fish species that act as climate change sentinels alongside protected species listed under the Washington Convention for endangered flora and fauna. Integrating scientific research, policy, and sustainable local development is crucial to safeguarding this unique coastal ecosystem. Ultimately, the UVS methodology and similar efforts are highlighted as essential tools for increasing environmental awareness, advancing scientific research, and supporting conservation efforts. Ongoing qualitative assessments are particularly important in fragile coastal areas threatened by human activities, pollution, and climate change.

## 1. Introduction

Biodiversity studies are essential for evaluating environmental quality and ecosystem integrity. In aquatic systems—whether freshwater, estuarine, or marine—metrics such as biological diversity, abundance, species tolerance, and community composition are widely used to assess ecosystem health [1]. Italy’s coastline stretches over 8300 km, with the Adriatic coast primarily characterized by sandy beaches and shallow waters, except for a few isolated rocky areas [2]. In the central Adriatic Sea (GSA17), notable features include Mount Conero and the small San Nicola Rock [3]. However, natural rocky formations are otherwise sparse on the Italian coastal line.

One exception is the Trabocchi Coast in Abruzzo, a rugged, steep shoreline renowned for its wild landscape, distinguished by cliffs and large landslides [4]. The region’s landscape has been shaped by selective erosion, driven by the interplay of marine geomorphic processes—such as Late Quaternary sea-level changes—and tectonic activity, which have molded the coastline and surrounding plateaus [4]. The Trabocchi Coast extends for approximately 55 km, between the mouths of the Foro and Trigno rivers, and it derives its name from the *trabocchi*, traditional wooden fishing structures anchored to rocks or reefs. These structures enabled fishing without the use of boats, and their origin in Abruzzo is believed to date back to the late 1600s when locals, unaccustomed to seafaring, repurposed a war machine known as the *trabucco* for fishing [5,6]. Today, these delicate wooden platforms have now been reinforced with durable materials like Robinia wood and iron. They feature a walkway that accommodates shifting tides and a winch system for lowering nets into the water. This fishing technique, relying on near-shore depths of 3–5 m, sustained the local economy until the early 1900s. In the early 20th century, their use resurged, with around 50 *trabocchi* documented in the Chieti coastal area [5]. Currently, *trabocchi* have mostly been converted into restaurants, shifting away from their traditional purpose, especially since the development of a coastal cycle path that has transformed the Trabocchi Coast into a popular tourist destination.

While tourism has brought economic benefits, it has also increased human pressure on this fragile coastline. The surge in accommodation facilities may outpace proper environmental planning, leading to challenges such as untreated sewage discharges, waste overloads, and the contamination of coastal waters via small streams [7]. Pleasure boats, attracted to the coast’s scenic beauty, also pose risks to the marine ecosystem, particularly to vulnerable seagrass meadows. Addressing these issues requires a comprehensive understanding of the coastal subaquatic biome, but current data remain scarce and fragmented. This is especially true for the infralittoral zone, where human activities directly interact with the marine ecosystem.

Although the Trabocchi Coast falls within GSA17 and is listed under the EU Habitat Directive as a protected area (1160—large shallow inlets and bays, 1170—reefs), there are limited data on its underwater biodiversity, particularly concerning fish, macroinvertebrates, and algae—key indicators used in biomonitoring [8]. Non-invasive observation methods like UVSs are particularly well-suited for studying protected species, including *Scyllarides latus* (Latreille, 1803), *Lithophaga lithophaga* (Linnaeus, 1758), *Sciaena umbra* (Linnaeus, 1758), *Ombrina cirrosa* (Linnaeus, 1758), and *Dentex dentex* (Linnaeus, 1758), the latter being a habitat fish bio-indicator [9]. Continuous monitoring is also critical for tracking the emergence of “climate fish”, species that serve as indicators of climate change [10]. Fish species are particularly valuable in signaling environmental changes due to their responsiveness to shifting climatic conditions, wide distribution, and ease of identification [11]. Ocean State Report 5 [12] highlights the strong linkage between changes in the South Adriatic hydrography—specifically salinity—and local biodiversity, with potential effects on commercially important fish species. Over the past two decades, the Adriatic Sea has seen the arrival of more than 20 non-native fish species, including Lessepsian migrants [13,14,15], and arthropoda from the Atlantic Ocean, such as the blue crab *Callinectes sapidus* (Rathbun, 1896), which have become increasingly common [16]. Monitoring the presence of new species can serve as an early warning, helping to protect native organisms from invasive species [17].

The primary objective of this long-term research project is to gather data on biodiversity and on rare, little-known, or difficult-to-find species, as well as update their distribution maps. In addition to identifying areas of interest, documenting sites where species are absent will contribute to a historical record of previous surveys [18]. Through a series of UVSs along the Trabocchi Coast, this study aims to provide a comprehensive, long-term picture of the infralittoral marine communities and their trends over time. The results will offer valuable insights into resident biodiversity and guide efforts to protect this unique ecosystem. Given the growing tourism industry and the increasing anthropogenic pressure on this narrow coastal strip, this research underscores the importance of sustainable ecotourism over a “visit-and-go” economy, promoting environmental stewardship alongside economic development.

## 2. Materials and Methods

### 2.1. Study Sites

**Figure 1:**
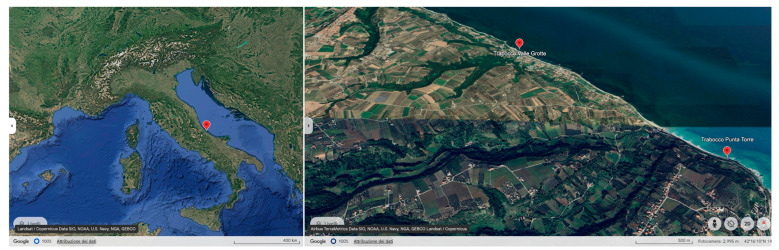
The location of the study areas along the mid-Adriatic A. Two locations were considered in this research: from 2011 to 2014, marine flora and fauna were recorded in the northern locality of Valle Grotte; from 2014 to date, the research has been carried out in the southern area of Rocca San Giovanni, both on the Chieti district, Abruzzo.

This study employed the UVS method through snorkeling and freediving across two sectors of the Trabocchi Coast along the Adriatic coastline in Chieti, Italy (Figure 1).

**Figure 2:**
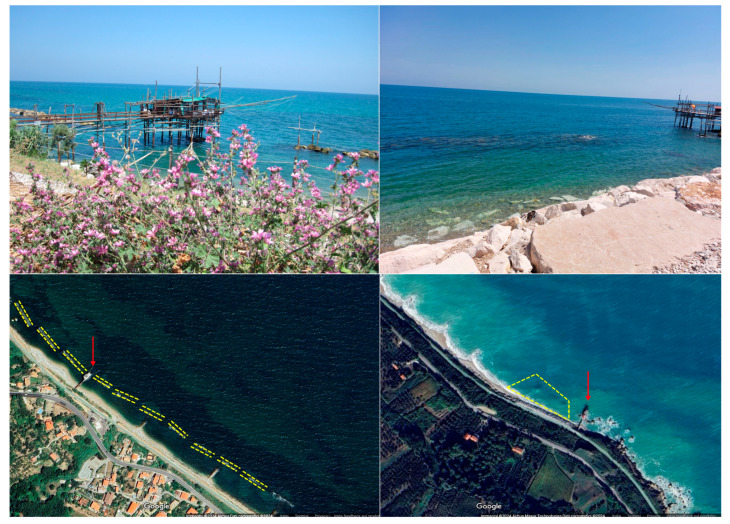
Top: a view of Trabocchi Valle Grotte (**left**) and Punta Torre (**right**), with the traditional structures with gangways extending into the water (Photo credits A. Arbuatti). Bottom: the geo-localization of the *trabocchi* (red arrows). The northernmost study area (**left**) consisted of underwater surveys on ten submerged reefs parallel to the coast (yellow dotted lines) for a total length of 648 m. The study area close to the Trabocco Punta Torre (**right**) consisted of video recording within the trapezoidal area that, from the coastline, reaches and exceeds the submerged reef to the left of the trabocco for a total area of 3300 square meters.

The northern survey area, located between Vallevò and Valle Grotte, 42°17′31″ N, 14°28′24″ E, spans the municipalities of San Vito Chietino and Rocca San Giovanni CH, Italy. Observations in this area were conducted between 2011 and 2014 [7] over ten submerged artificial reefs, which run parallel to the coastline for a total length of 648 m (Figure 2, left). The second survey site, Punta Torre in Rocca San Giovanni, 42°16′26″ N, 14°29′95″ E, has been monitored continuously since 2015 (Figure 2, right). This site has a trapezoidal shape, with its longer base aligned with the shoreline, and a shorter base consisting of artificial and biogenic reefs. These reefs extend 16 m in width, creating a total surveyed area of approximately 3300 m^2^.

**Figure 3:**
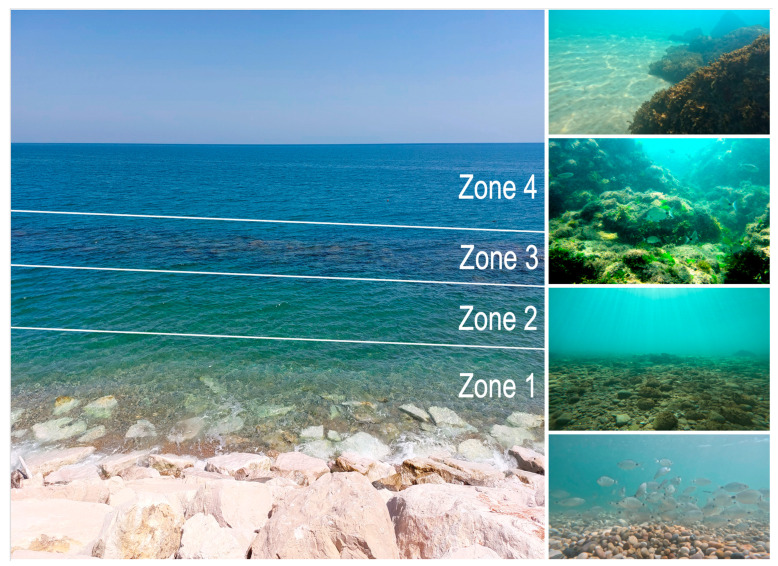
The picture shows the typical pattern of the Trabocchi Coast. Pictures of zones 1–4 from the outside (**left**). (**Right**) pictures of zones 1–4 (from bottom to top) taken underwater. From zone 1 (shoreline) to zone 4 (open sea), different bottom areas can be found, each one with a specific substrate and depth. In a handful of meters, the coast goes from pebbles to sand, and to cliffs at approximately 5 m depth. This is due to the geological evolution of the zone, which represents a unique distinctive trait of the central part of the Frentane coast (Photo credit A. Arbuatti).

As illustrated in Figure 3, the benthic environment across both coastal stretches is characterized by significant topographic heterogeneity. The zones identified are as follows:

- **Zone 1**: medium-sized rocky pebbles near the shoreline, extending approximately 3–4 m offshore.
- **Zone 2**: transition substrate with sand, mud, gravel, scattered rocks, and small reef fragments, extending 10–30 m offshore.
- **Zone 3**: geogenic and biogenic reefs running parallel to the coastline.
- **Zone 4**: deeper offshore areas characterized by fine, nearly muddy sand and additional underwater reefs, whose distribution is only partially mapped to date.

The depth measurements in these zones range from 0 to over 500 cm further offshore, with a slight rise in the seabed as it approaches the coastal *trabocco*. Historical depth data for the northernmost study area Trabocco Valle Grotte were unavailable due to modifications following the installation of new artificial brush reefs after 2015.

Diver-operated stereo video and photographic surveys were conducted annually from 2011 to 2024, typically between June and August. Some surveys extended into September, depending on weather conditions, though no data were collected in June 2021 due to COVID-19 restrictions. A total of 267 snorkeling and free-diving sessions were performed, averaging 20 sessions per year. Each session, lasting approximately 60 min, was conducted by a single operator (AA) in the morning to minimize wave interference. Swimming speed was maintained at 1–3 m per minute. A second operator (PL, ADS) was present during each session to provide technical or emergency support.

During each session, the operator entered the water from the shore, reached the reef, and performed two round trips along both sides of the reef. This was followed by free movement between the shoreline and the reef. To minimize the risk of damage to the reef, free diving was performed at a sufficient distance from the reef. In total, 733 short videos and 2596 photographs were captured and stored on external hard drives for analysis.

### 2.2. Equipment and Tools

The digital tools used for recording marine life evolved over the course of the study. Various cameras and devices were employed, including a Canon Powershot D10 (Canon Inc., Öita, Japan), a GoPro7 (GoPro Inc., San Mateo, CA, USA), a Fujifilm XP (Fujifilm Holdings Corporation, Minato Tokyo, Japan), an AKASO7 (AKASO, Fredrick, MD, USA), and a GoPro11 (GoPro Inc., San Mateo California, USA). All recorded videos and photographs were transferred to hard drives for subsequent analysis. Software tools used for analysis included Google Picasa 3.9, Photo (Windows 7and 8), Windows Clipchamp (Windows 11), and Windows Media Player (Windows 11). These tools, particularly the frame-by-frame functionality, aided in identifying phenotypic traits and supported the taxonomic identification of species. This was crucial for addressing challenges such as poor weather conditions, image quality, and animal behavior during observations.

In addition, snorkeling equipment included short fins to prevent reef damage and wide-vision masks for enhanced underwater visibility, facilitating smoother maneuverability in confined areas.

### 2.3. Fish Identification

The videos and photographs collected from 2011 to 2024 were randomly distributed among the authors to identify fish fauna along the Trabocchi Coast. Three evaluators—AA, ADS, and PL—participated in the identification process, each with distinct areas of expertise: zoology, fish medicine, and aquarium management; environmental biology, and ethology, respectively. The classification scheme used to assess concordance between the evaluators was developed based on the Louisy manual 2022 [19] and the FishBase electronic database [20], covering 21 fish species commonly found in the Adriatic Sea.

To ensure consistency in species identification, the key characteristics of each species were repeatedly reviewed and discussed in various contexts. Krippendorff’s Alpha, a statistical measure well-suited for studies involving multiple raters, was employed to evaluate inter-rater reliability [21]. This method is robust for handling different numbers of raters, variable sample sizes, and missing data, making it ideal for this study [22]. The inter-rater reliability results, calculated across different levels of Krippendorff’s Alpha, are presented in Table 1.

### 2.4. Statistical Analysis

The statistical analyses on raters’ concordance were conducted using JASP version 0.18.3 2024, open-source statistical software [23]. Data were analyzed to compute Krippendorff’s Alpha with a 95% confidence interval, based on 1000 bootstrap iterations.

## 3. Results

The concordance analysis yielded a reliability coefficient that quantifies the degree of agreement among the raters beyond what would be expected by chance (Table 2).

The computed Krippendorff’s Alpha was α = 0.90 (SE = 0.04, 95% C.I. = 0.82–0.97), indicating strong inter-rater reliability. As per Krippendorff [21], the threshold for a satisfactory level of reliability is 0.80, confirming that the achieved reliability is well within the acceptable range [22].

Through the analysis of the collected images and videos, a total of 46 fish species, categorized into 18 families, were identified within the two specific stretches of the Trabocchi Coast examined (Table 3).

**Figure 4:**
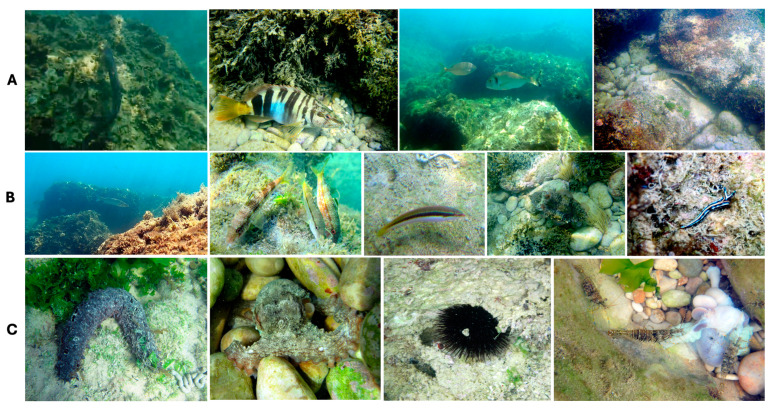
A few specimens recorded on the Trabocchi Coast. From left to right: Line (**A**): *Conger conger, Serranus scriba*, *Sparus aurata* and *Sarpa salpa*, *Sphyraena sphyraena*; Line (**B**): *Dicentrarcus labrax*, *Mullus surmuletus*, *Coris julius*, *Sepia officinalis*, *Thuridilla hopei*; Line (**C**): *Holoturia tubulosa*, *Octopus vulgaris*, *Arbacia lixula*, *Palaemon elegans*. More species are included in the supplementary files (Photo credits: A. Arbuatti).

All identified species belong to the superclass Osteichthyes and the class Actinopterygii. Some fish larvae, however, could not be classified at the species level, based on the videos or photographs. These were identified as belonging to the Atherinidae and Carangidae families. Additionally, certain species from the Mugilidae family that were difficult to classify were excluded from the report. Some species are included in Figure 4; more species can be found in the Supplementary Files.

### 3.1. Invertebrates

The footage also enabled the classification of 35 invertebrate species, including both sessile and non-sessile organisms. These species span multiple phyla: 14 species from Mollusca, eight from Arthropoda Malacostraca and Thecostraca, seven from Cnidaria (Anthozoa, Hexacorallia, Hydrozoa, and Scyphozoa), three from Echinodermata (Echinoidea and Holothuroidea), three from Porifera (Demospongiae), and one from Chordata (Ascidiacea), as shown in Table 4. Some species are included in Figure 4, more can be found in the Supplementary Files.

### 3.2. Producers

**Figure 5:**
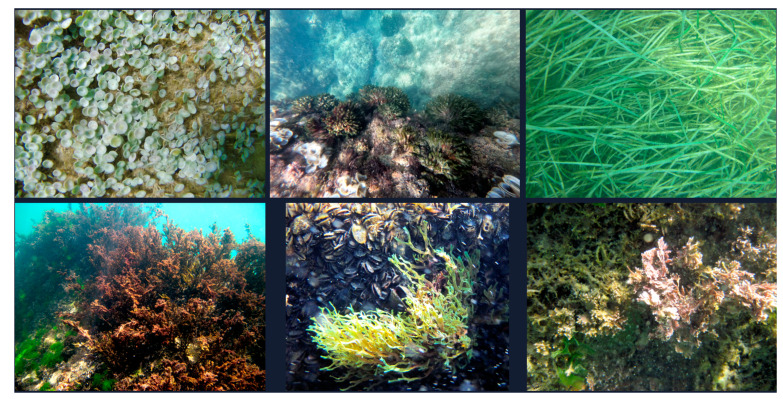
Various species of algae and plants (producers) are found among the cliffs of the Trabocchi Coast at the two investigation sites. From left to right and from top to bottom: *Acetabularia acetabulum*, *Codium fragile*, *Cimodocea nodosa Ucria*, *Cystoseira adriatica* and *Ulva lactuca*, *Dictyota dichotoma*, *Ellissolandia elongata* (Photo credits A. Arbuatti).

For a comprehensive analysis of the trophic chain, the primary producers present in the research areas, including marine algae and plants, were also recorded. Using online databases, we identified and classified nine algal species across the phyla Chlorophyta, Heterokontophyta, and Rhodophyta, along with one seagrass species from the class Tracheophyta (Table 5). Some of the species are shown in Figure 5, while additional species are provided in the Supplementary Files.

## 4. Discussion

The coastal area of Abruzzo extends for approximately 133 km, with its southern section, between the river mouths of the Foro and Trigno (Chieti,) known as the “Trabocchi Coast”. This stretch is mainly characterized by rocky seafronts, small bays, inlets, and submerged cliffs. Together with the northern marine area of Mount Conero (Ancona) and San Nicola Rock (Ascoli Piceno), it represents one of the few natural rocky portions of the western Adriatic coast (GSA 17), in the mid-Adriatic region [3]. The bedrock of the Trabocchi Coast is composed of siliciclastic deposits from the Plio-Pleistocene marine successions. This area features clayey-sandy and conglomeratic deposits, with a marine foredeep affected by significant lowering during the Pliocene and Quaternary periods [24]. The coastline is shaped continuously by marine currents, wind, wave motion, and tides, as well as by human interventions, creating a unique ecological interconnection between the terrestrial and marine environments of the central Italian Adriatic. The sites investigated are protected under the EU Habitat Directive (Council Directive 92/43/EEC) as Habitat 1170 (reefs) and Habitat 1160 (large shallow inlets and bays).

The Trabocchi Coast is particularly dynamic, both geologically and marine-wise, making it vulnerable to endogenous and exogenous phenomena [25]. It is common to see cliffs and exposed rocky portions along the coast uninterruptedly connected to the inland hilly mountainous terrain. Coastal rocky habitats support diverse necto-benthic fish communities due to the availability of shelter and food [26]. However, coastal ecosystems, among the most productive on the planet, are also highly vulnerable to human activities like fishing, pollution, urbanization, and boat anchoring. These activities negatively impact habitats such as *Posidonia oceanica* (Delile, 1813) meadows, critical to Mediterranean marine biodiversity [27,28]. Furthermore, anthropogenic factors, like boat moorings, contribute to the spread of invasive species [29], compounding the problem. The challenge of estimating the recovery time for damaged marine phanerogams globally adds to the complexity of protecting these ecosystems [29].

Despite its relatively short coastline compared to other regions in the mid- and northern Adriatic, the marine ecosystems of the Chieti southern areas have been underexplored. Most research is focused on the northern coast, particularly around the Marine Protected Area of the Torre del Cerrano [30,31,32,33,34]. Moreover, these studies largely rely on netting techniques, which can provide quantitative data but often result in the death of marine specimens and fail to capture smaller, cryptic species like combtooth blennies, which require visual census methods [35,36]. In contrast, studies on the Trabocchi Coast remain scarce, despite its environmental significance, as confirmed by its inclusion in the Habitat Directive. Limited surveys on marine biodiversity in the shallow water of this area have been conducted [7,37,38,39]. Our research, spanning 14 years, is the longest and most comprehensive study conducted on this part of the Adriatic coast. It reveals much richer and more diverse fauna than previously documented.

We recorded 46 fish species with 2 unclassified ones, a significant finding for the region and one of the highest recorded in the Italian Adriatic infralittoral zone. Our study confirms the Trabocchi Coast as a nursery for fish populations, essential for renewing adult fish stocks, which highlights the importance of protecting these coastal areas [40,41,42]. Notably, species of economic importance, such as those from the *Diplodus* genus (*D. puntazzo*, *D. vulgaris*, and *D. sargus*), *Dicentrarchus labrax*, *Sparus aurata*, and *Sciaena umbra*, were observed. Moreover, 67.4% of the species recorded are listed as bio-indicators of ecological quality by EU Directive 2024/721. Among these species, five (*Coris julis*, *Serranus cabrilla*, *Serranus scriba*, *Pomatomus saltatrix*, *Sarpa salpa*, and *Thalassoma pavo*) are considered “climate fish”, validated indicators of climate change [10].

Primary producers like seagrasses and macroalgae, alongside tertiary consumers such as sea bass and barracuda, indicate a healthy trophic chain. Additionally, we documented the presence of species of regulatory interest, such as *Lithopaga lithopaga* and *Cladocora caespitosa,* both listed in CITES Appendix II, alongside alien species like *Rapana venosa* and the harmful dinoflagellate *Ostreopsis ovata*, which poses public health risks. Compared to other Adriatic reef studies (see Table S1 in Supplementary Files), our findings demonstrate that the Trabocchi Coast harbors rich biodiversity, positioning it as a key Adriatic ecosystem.

In terms of methodology, we employed a qualitative UVS using natural-light video recordings. This non-destructive approach enables the recording of fish fauna with minimal environmental impact, making it ideal for habitats with varied substrates, such as those found along the Trabocchi Coast of the Adriatic Sea [43,44]. UVSs are particularly effective at capturing cryptic or elusive species often missed by traditional transect methods, including shy predators, like larger fish that tend to avoid divers, and small, concealed species, such as blennies hidden under rocks [11,45,46]. Unlike conventional transects, which may underestimate these species, operator-driven video recording allows divers to explore habitats freely, without the constraints of transect width, thereby enabling comprehensive documentation in heterogeneous and patchy environments like rocky and sandy seabed [47,48]. Our extended survey sessions, each lasting approximately one hour, enhanced the detection of small and cryptic species, providing valuable qualitative data for long-term ecological studies.

Recently, underwater drones have gained popularity in research but introduce disruptive noise and lighting. In narrow coastal areas, between rocks or in shallow waters, drones face limited maneuverability and cannot accurately census species living in crevices between rocks. Occasionally, recreational snorkeling or diving activities contribute data on the presence of marine species, from algae to fish (citizen science). However, such contributions must be approached cautiously. Summer tourists, despite their passion for marine environments, may lack the ability to accurately identify species they encounter during a brief, once-a-year recreational swim, as highlighted by the “Sea Sentinel” project’s final report. Between 2017 and 2022, this project in Abruzzo collected 36 survey sheets, all deemed unusable [38]. Instead, citizen science efforts should be supported through the training of competent individuals, such as with scuba diving schools, aquatic veterinarians, and biologists, who can accurately conduct underwater censuses.

Supporting the value of our research, a 2023 review of non-destructive and non-extractive UVS methods in the Mediterranean Sea revealed that studies conducted in the water column are scarce due to the significant effort required to record even a small number of specimens [44]. While the UVS method we used does have limitations, these are mitigated by the longitudinal nature of our study, which applied the technique over a wide temporal scale in the same coastal areas year after year. As a result, biases related to the survey’s timing (summer mornings) have been minimized, allowing for the observation of both common and cryptic species, as well as those with unique (solitary) behavioral traits. Long-term and regular species inventories are vital for monitoring habitat responses to changes, in line with the Habitat Directive 92/43/CEE. Annual surveys, for instance, provide valuable opportunities to assess biotic responses to climate change using a limited number of reliable bioindicators [10].

One limitation of UVS is that it may overemphasize the importance of rare species while underrepresenting abundant ones. However, it remains highly effective for observing marine species’ movements within crevices, allowing for detailed post-survey video analysis [48,49,50]. This method can yield better results for qualitative censuses than others by leaving a record that local populations can use to assess the status of complex ecosystems along the Trabocchi Coast, just a few meters from the shoreline. These coastal areas are significant hot spots for human activities and the damage they can cause. Therefore, proper management and control of anthropogenic activities are crucial, ranging from enforcing regulations on underwater sport fishing often conducted without adhering to articles 129–130 of Presidential Decree 1639/68 to preventing the anchoring of pleasure boats on seagrass beds [7,51]. The increasing development of this part of the Abruzzo coast as a tourist destination, albeit an eco-sustainable one, raises concerns that the particularly fragile landscape of the Trabocchi Coast may not withstand a fast-growing, hit-and-run form of tourism. The rapid expansion of tourism infrastructure, such as residential settlements, beach resorts, campsites, and kiosks, alongside moorings and noise from tourist boats, and excessive sport fishing, could severely impact this already endangered ecosystem. The situation can be exacerbated by obsolete sewage treatment facilities, which may not cope with the increased load resulting from intensified coastal use. Although this scenario has not yet reached the levels seen in northern Abruzzo’s more tourism-driven areas [52], only the careful environmental management of the Trabocchi Coast can foster a sustainable, alternative local development model. The fragility of the Trabocchi coastal area has also been highlighted by ISPRA, which identified flood and landslide hazards along much of the coastline without sufficient mitigation and adaptation measures [53]. In addition to implementing water purification measures to reduce pollution and eutrophication in coastal waters, global awareness is essential to mitigate the impacts of climate change. Various studies suggest a ‘meridionalization’ of the Adriatic Sea, with projections indicating a surface temperature increase of 1.5 °C by 2040, accompanied by rising sea levels and increased salinity [54,55,56,57]. These environmental shifts may facilitate the spread of invasive alien species, which pose a serious threat to native species, as seen in the case of the Atlantic blue crab [58].

## 5. Conclusions

Scientific, qualitative studies of aquatic ecosystems are essential for regions like the Trabocchi Coast, where the economy relies on its natural and cultural heritage. In addition to the iconic *trabocchi* fishing platforms, local economies benefit from small-scale inshore fishing, niche ecotourism, and snorkeling. To safeguard the coastal ecosystem, it is essential to promote local economic development through structural investments while ensuring the protection of biodiversity. Furthermore, scientific research and educational initiatives should be encouraged [59] in the form of information boards, mini-guides, lessons for school groups during practical teaching activities, and small museums dedicated to the history of the ancient *trabocchi*, all alongside a sustainable approach to niche ecotourism. The growth of ecotourism education, along with stronger recommendations for the use of local fish resources in catering, regional support, international funding opportunities, and increased participation in tourism eco-certification and eco-label programs, can collectively foster strong conservation behaviors among both locals and visitors [60].

However, the effective protection of the area, including marine environments, waters, seabeds, and adjacent coastlines, can only be achieved once the technical-investigatory process is complete. These regions are of great interest due to their natural, geomorphological, physical, and biochemical characteristics, particularly concerning marine and coastal flora and fauna, as well as their scientific, ecological, cultural, educational, and economic importance. This is especially true for the Adriatic region, as underscored by the final report on the National Biodiversity Strategy 2011–2020 from the Ministry of the Environment [61], which laid the groundwork for the 2023 National Biodiversity Strategy.

Monitoring fish populations is critical because understanding species variability in a community over time allows local governments to take legal action to protect fragile ecosystems. Only by understanding the local aquatic biodiversity can communities become aware of (i) biodiversity loss, (ii) species threatened by overfishing, (iii) pollution, or (iv) climate change impacts. Additionally, this approach helps safeguard fish populations by raising awareness of the positive effects of policy decisions, such as increased species diversity, population numbers, and fish size—indicators of a balanced ecosystem recovering from pressures like overfishing or pollution [62].

Achieving these goals aligns with the definition of sustainability outlined by the Brundtland Commission in 1987: “*meeting the needs of the present without compromising the ability of future generations to meet their own needs*”.

## Figures and Tables

**Table 1:** Level of agreement among different raters, according to Krippendorff [21].

Krippendorff’s Alpha Value	Interpretation
α = 1	perfect agreement
α ≥ 0.80	satisfactory level of agreement
α = 0.67–0.79	moderate agreement
α < 0.67	poor agreement
α = 0	no agreement
α < 0	total disagreement

**Table 2:** Results of the K-alpha for inter-raters’ agreement.

Krippendorff’s Alpha				
			95% CI	
Method	Krippendorff’s alpha	SE	Lower	Upper
Nominal	0.90	0.04	0.82	0.97

**Table 3:** Fish species whose presence was documented in the research fields.

Family	*Genus*	*Specie*	*Author*	*2011–2014*	*2015–2024*	*Age*	*Zone*	*Commercial Interest*
**Atherinidae**	*Atherina*	*A.* spp.		✓	✓	*A*	*2, 3*	✓
**Belonidae**	*Belone*	*B. belone*	Linnaeus, 1761	✓	✓	*J/A*	*1A, 2J*	✓
**Blennidae**	*Aidablennius*	*A. sphynx*	Valenciennes, 1836	✓	✓	*A*	*1, 3*	
	*Lipophrys*	*L. dalmatinus*	Steindachner and Kolombatović, 1883	✓		*A*	*3*	
		*L. capone*	Linnaeus, 1758		✓	*J*	*3*	
	*Microliphoprys*	*M. canevae*	Vinciguerra, 1880	✓	✓	*J/A*	*3*	
	*Parablennius*	*P. gattorugine*	Linnaeus, 1758	✓	✓	*A*	*3*	
		*P. incognitus*	Bath, 1968	✓	✓	*J/A*	*3*	
		*P. rouxi*	Cocco, 1833		✓	*J*	*3*	
		*P. sanguinolentus*	Pallas, 1814	✓	✓	*A*	*3*	
	*Salaria*	*S. pavo*	Risso, 1810	✓	✓	*A*	*1, 3*	
**Carangidae**	*Trachinotus*	*T. ovatus*	Linnaeus, 1758	✓	✓	*J*	*1, 2*	✓
	*Lichia*	*L. amia*	Linnaeus, 1758		✓	*J*	*2,* 3	✓
**Congridae**	*Conger*	*C. conger*	Linnaeus, 1758		✓	*J*	*3*	✓
**Gobiidae**	*Gobius*	*G. cobitis*	Pallas, 1814	✓	✓	*A*	*3*	
	*Pomatoschiustus*	*P.* spp.		✓	✓	*J*	*1*	
**Labridae**	*Symphodus*	*S. melops*	Linnaeus, 1758	✓	✓			
		*S. roissali*	Risso, 1810	✓	✓	*J/A*	*2,3*	
		*S. tinca*	Linnaeus, 1758	✓	✓	*J/A*	*3*	
	*Coris*	*C. julis*	Linnaeus, 1758	✓	✓	*J/A*	*2,* 3	
	*Thalassoma*	*T. pavo*	Linnaeus, 1758		✓	*J*	*3*	
**Moronidae**	*Dicentrarchus*	*D. labrax*	Linnaeus, 1758	✓	✓	*J/A*	*2,* 3	✓
**Mugilidae**	*Liza*	*L. aurata*	Risso, 1810	✓	✓	*A*	*2,* 3	✓
	*Chelon*	*C.ramada*	Risso, 1827		✓	*A*	*1, 2, 3*	✓
**Mullidae**	*Mullus*	*M. surmuletus*	Linnaeus, 1758	✓	✓	*J/A*	*1, 2, 3*	✓
**Phycidae**	*Phycis*	*P. phycis*	Linnaeus, 1766	✓ *dead*		*A*	*3*	✓
**Pomacentridae**	*Chromis*	*C.chromis*	Linnaeus, 1758		✓	*J*	*3*	
**Pomatomidae**	*Pomatomus*	*P. saltatrix*	Linnaeus, 1766	✓ *bites on fishes*	✓ *bites and carcass*	*J/A*	*3 and ashore*	✓
**Sciaenidae**	*Sciaena*	*S. umbra*	Linnaeus, 1758		✓	*J*	*3*	✓
**Scorpaenidae**	*Scorpaena*	*S. porcus*	Linnaeus, 1758		✓	*J/A*	*3*	✓
**Serranidae**	*Serranus*	*S. cabrilla*	Linnaeus, 1758		✓	*J*	*2,* 3	
		*S. hepatus*	Linnaeus, 1758		✓	*J*	*1*	
		*S. scriba*	Linnaeus, 1758	✓	✓	*A*	*3*	✓
**Sparidae**	*Boops*	*B. boops*	Linnaeus, 1758	✓	✓	*J/A*	*2 school, 3*	
	*Dentex*	*D. gibbosus*	Rafinesque, 1810		✓	*J*	*3*	✓
	*Diplodus*	*D. annularis*	Linnaeus, 1758	✓	✓	*J/A*	*3*	✓
		*D. puntazzo*	Walbaum, 1792	✓	✓	*J/A*	*3*	✓
		*D. sargus*	Linnaeus, 1758	✓	✓	*J/A*	*3*	✓
		*D. vulgaris*	Geoffroy Saint-Hilaire, 1817	✓	✓	*J/A*	*2,* 3	✓
	*Lithognathus*	*L. mormyrus*	Linnaeus, 1758		✓	*A*	*2*	✓
	*Oblada*	*O. melanurus*	Linnaeus, 1758	✓	✓	*J/A*	*1, 2, 3*	✓
	*Sarpa*	*S. salpa*	Linnaeus, 1758	✓	✓	*J/A*	*2, 3*	✓
	*Spondyliosoma*	*S. cantharus*	Linnaeus, 1758	✓	✓	*J*	*3*	✓
	*Pagrus*	*P. pagrus*	Linnaeus, 1758	✓	✓	*J*	*3*	✓
	*Sparus*	*S.aurata*	Linnaeus, 1758	✓	✓	*J/A*	*1, 2, 3*	✓
**Sphyraenidae**	*Sphyraena*	*S. sphyraena*	Linnaeus, 1758	✓		*J*	*2*	✓
**Fish fry**								
**Atherinidae**	Atherinae sp.	*Atherinae* sp.		✓	✓	J few mm	*2*, 3	?
**Carangidae**	spp.	*2 fish larvae among R. pulmo arms*			✓	J few mm	3	?

Data from 2011 to 2014 refer to the first survey area, located between Vallevò and Valle Grotte, 42°17′31″ N, 14°28′24″ E; data from 2015 to present refer to the second study site, Punta Torre in Rocca San Giovanni, 42°16′26″ N, 14°29′95″ E. ✓: present in the area. Age categories; A, adult; J, juvenile. Zone 1 comprises underwater pebbles near the shoreline; Zone 2 is made of transitional substrate with sand, mud, gravel, scattered rocks, and small reef fragments; Zone 3 consists of the reef, and Zone 4 is the open sea. Commercial interest follows the definition of Fishbase.org [20]. Some species listed in Table 3 are included in Figure 4.

**Table 4:** Species of invertebrates belonging to different phyla whose presence was recorded in both study areas.

Phyla	Class	Genus	Species	Author	Zone
Arthropoda	Malacostraca	*Clibanarius*	*C. erythropus*	Latreille, 1818	3
		*Eriphia*	*E. verrucosa*	Forskål, 1775	3
		*Maja*	*M. squinado*	Herbst, 1788	3
		*Isopoda*			2, 3
		*Pachygrapsus*	*P. marmoratus*	Fabricius, 1787	1, 3
		*Palaemon*	*P. elegans*	Rathke, 1836	1
		*Unclassified*	*free floating larvae-juvenile*		3
	Thecostraca	*Balanus*	*B.* spp.		3
Cnidaria	Anthozoa	*Anemonia*	*A. sulcata*	Pennant, 1777	2, 3
		*Actinia*	*A. equina*	Linnaeus, 1758	3
		*Cladocora*	*C. Caespitosa*	Linnaeus, 1767	3
	Hexacorallia	*Aiptasia*	*A. diaphana*	Rapp, 1829	3
	Hydrozoa	*Pennaria*	*P. disticha*	Goldfuss, 1820	3
	Scyphozoa	*Cotylorhiza*	*C. tuberculatadead ^*	Macri, 1778	^
		*Rhizostoma*	*R. pulmo*	Macri, 1778	2, 3
Echinodermata	Echinoidea	*Arbacia*	*A. lixula*	Linnaeus, 1758	3
		*Paracentrotus*	*P. lividus*	Lamarck,1816	3
	Holothuroidea	*Holothuria*	*H. tubulosa*	Gmelin, 1788	2
Mollusca	Bivalvia	*Crassostrea*	*C. gigas*	Thunberg, 179	3
		*Lithophaga*	*L. litophaga*	Linnaeus, 1758	3
		*Mytilus*	*M. galloprovincialis*	Lamarck,1819	3
		*Ostrea*	*O. edulis*	Linnaeus 1758	3
	Cefalopoda	*Octopus*	*O. vulgaris*	Cuvier, 1797	1, 2, 3
		*Sepia*	*S. officinalis*	Linnaeus, 1758	2, 3
	Gastropoda	*Aplysia*	*A. depiilans*	Gmelin, 1791	2, 3
		*Hexaplex*	*H. trunculus*	Linnaeus, 1758	3
		*Ocinebrina*	*O. edwardsii*	Payraudeau, 1826	3
		*Patella*	*P.* sp.		3
		*Phorcus*	*P. turbinatus*	Born, 1778	1, 2, 3
		*Rapana*	*R. venosa*	Valenciennes, 1846	3
		*Thuridilla*	*T. hopei*	Verany, 1853	3
Porifera	Demospongiae	*Aplysina*	*A. aerophoba*	Nardo, 1833	2, 3
		*Chondrosia*	*C. reniformis*	Nardo, 1847	3
		*Cliona*	*C.* spp.	Grant, 1826	3
Chordata	Ascidiacea	*Ascidie*		Heller, 1877	3

^ Beached specimen.

**Table 5:** Algae and plants species whose presence was recorded in both study areas.

Phyla	Class	Genus	Species	Author	Zone
Chlorophyta	Ulvophyceae	*Codium*	*C. fragile*	Hariot, 1889	3
		*Ulva*	*U. lactuca*	Linnaeus 1753	2, 3
		*Acetabularia*	*A. acetabulum*	Linnaeus	2
Heterokontophyta	Phaeophyceae	*Padina*	*P. pavonica*	Linnaeus	2, 3
		*Cystoseira*	*C. adriatica*	Sauvageau, 1912	3
		*Dictyopteris*	*D. polypodioides*	De Candolle J.V. Lamoroux, 1809	3
		*Dictyota*	*D. dichotoma*	Hudson J.V. Lamoroux, 1809	3
Rhodophita	Florideophyceae	*Ellisolandia*	*E. elongata*	Hind and Saunders, 2013	3
		*Halymenia*	*H. floresii Clemente*	Agardh, 1817	3
Tracheophyta	Magnoliopsida	*Cymodocea*	*C. nodosa Ucria*	Ascherson, 1870	2

## Data Availability

The original contributions presented in the study are included in the article/Supplementary Material, further inquiries can be directed to the corresponding author.

## Supplementary Materials

The following supporting information can be downloaded at https://www.mdpi.com/article/10.3390/ani14233469/s1, Table S1: Monitoring of marine species in the western Adriatic outside Abruzzo’s coastlines. Zenodo: https://doi.org/10.5281/zenodo.14173294, (registered on 16 November 2024) “Long-term ecosystem monitoring along the Trabocchi Coast (Chieti, Italy): insights from underwater visual surveys (2011–2024)”. References [63,64,65,66,67,68,69,70,71,72,73] are cited in the supplementary materials.

## References

[1] O’Brien A., Townsend K., Hale R., Sharley D., Pettigrove V. (2016). How Is Ecosystem Health Defined and Measured? A Critical Review of Freshwater and Estuarine Studies. Ecol. Indic..

[2] ISPRA (2024). Linea di Costa in Italia: 120 km² di Superficie Complessiva di Spiagge oltre Due Terzi Nelle Regioni del Sud e Nelle Isole Maggiori. https://www.isprambiente.gov.it/it/istituto-informa/comunicati-stampa/anno-2024/.

[3] Minelli A., Ferrà C., Spagnolo A., Scanu M., Tassetti A.N., Ferrari C.R., Fabi G. (2020). The ADRIREEF Database: A Comprehensive Collection of Natural/Artificial Reefs and Wrecks in the Adriatic Sea. Earth Syst. Sci. Data Discuss..

[4] Miccadei E., Piacentini T., Buccolini M. (2017). Long-Term Geomorphological Evolution in the Abruzzo Area, Central Italy: Twenty Years of Research. Geol. Carpath..

[5] D’Uva D., D’Uva C. (2018). I Trabucchi. Macchine da Pesca Tradizionali tra Geometria e Tecnologia.

[6] Mascitelli A., Prestileo F., Stella E.M., Aruffo E., López Campos L.I., Federico S., Dietrich S. (2023). Impact of Climate Change on the “Trabocchi Coast,” Italy: The Trabocco Turchino Case Study. Sustainability.

[7] Arbuatti A. Studio Qualitativo della Fauna Subacquea Associata alle Barriere Artificiali Sommerse nella Costa dei Trabocchi CH Mediante Underwater Visual Census. Proceedings of the 1st International Meeting of the Italian Society of Exotic Animal Veterinarians.

[8] La Mesa G., Tunesi L., La Mesa G., Paglialonga A., Tunesi L. (2019). Scyllarides latus Latreille, 1802 (Magnosa). Handbooks for Monitoring Species and Habitats of Community Interest (Council Directive 92/43/EEC and Directive 09/147/CE in Italy: Marine Environment).

[9] Catalano B., Penna M., Riccato F., Fiorin R., Franceschini G., Antonini C., Franzoi P., Catalano B., Penna M., Cicero A.M. (2017). Manuale per la Classificazione dell’Elemento di Qualità Biologica “Fauna Ittica” Nelle Lagune Costiere Italiane—Applicazione dell’Indice Nazionale HFBI Habitat Fish Bio-Indicator ai Sensi del D. Lgs 152/2006.

[10] Azzurro E., Ballerini T., Antoniadou C., Aversa G.D., Souissi J.B., Blašković A.J. (2022). ClimateFish: A Collaborative Database to Track the Abundance of Selected Coastal Fish Species as Candidate Indicators of Climate Change in the Mediterranean Sea. Front. Mar. Sci..

[11] Rocculi A. (2021). Standardized Methodologies to Monitor Climate Change Effects on the Ichthyofauna of the Conero Area. Ph.D. Thesis.

[12] von Schuckmann K., Le Traon P.Y., Smith N., Pascual A., Djavidnia S., Gattuso J.P., Zupa W. (2021). Copernicus Marine Service Ocean State Report, Issue 5. J. Oper. Oceanogr..

[13] Dulčić J., Dragičević B., Grgičević R., Lipej L. (2011). First Substantiated Record of a Lessepsian Migrant—The Dusky Spinefoot, *Siganus luridus* (Actinopterygii: Perciformes: Siganidae), in the Adriatic Sea. Acta Ichthyol. Piscat..

[14] Dulčić J., Bello G., Dragičević B. (2020). *Bregmaceros nectabanus* Whitley, 1941 (Teleostei: Bregmacerotidae), a New Lessepsian Migrant in the Adriatic Sea. BioInvasions Rec..

[15] Kamberi E., Beqiri K., Luli K., Bakiu R. (2022). Tracking Changes in Fish Diversity in the South-Eastern Adriatic Sea (Albania) Based on Local Ecological Knowledge. Croat. J. Fish..

[16] Mancinelli G., Bardelli R., Zenetos A. (2021). A Global Occurrence Database of the Atlantic Blue Crab *Callinectes sapidus*. Sci. Data.

[17] Tsirintanis K., Azzurro E., Crocetta F., Dimiza M., Froglia C., Gerovasileiou V., Katsanevakis S. (2022). Bioinvasion Impacts: Biodiversity, Ecosystem Services, and Human Health in the Mediterranean Sea. Aquat. Invasions.

[18] La Mesa G., Paglialonga A., Tunesi L. (2019). Handbooks for Monitoring Species and Habitats of Community Interest (Council Directive 92/43/EEC and Directive 09/147/CE in Italy: Marine Environment).

[19] Louisy P., Trainito E. (2022). Guida all’Identificazione dei Pesci Marini d’Europa e del Mediterraneo.

[20] Froese R., Pauly D. (2024). FishBase.

[21] Krippendorff K. (2019). Content Analysis: An Introduction to Its Methodology.

[22] Marzi G., Balzano M., Marchiori D. (2024). K-Alpha Calculator–Krippendorff’s Alpha Calculator: A User-Friendly Tool for Computing Krippendorff’s Alpha Inter-Rater Reliability Coefficient. MethodsX.

[23] JASP Team (2024). JASP.

[24] Miccadei E., Carabella C., Paglia G. (2021). Morphoneotectonics of the Abruzzo Periadriatic Area, Central Italy: Morphometric Analysis and Morphological Evidence of Tectonics Features. Geosciences.

[25] Stoppa F. (2010). Geologia e Problematiche Ambientali della Costa Teatina. Riv. Abruzz..

[26] Molinari A. (2005). Fish Community Associated with Shallow Beachrock Rocky Reef of Ligurian Sea (NW Mediterranean). Cybium.

[27] Milazzo M., Badalamenti F., Ceccherelli G., Chemello R. (2004). Boat Anchoring on *Posidonia oceanica* Beds in a Marine Protected Area (Italy, Western Mediterranean): Effect of Anchor Types in Different Anchoring Stages. J. Exp. Mar. Biol. Ecol..

[28] Carreño A., Lloret J. (2021). Environmental Impacts of Increasing Leisure Boating Activity in Mediterranean Coastal Waters. Ocean Coast. Manag..

[29] Tiralongo F., La Mesa G., De Mendoza F.P., Massari F., Azzurro E. (2021). Underwater Photo Contests to Complement Coastal Fish Inventories: Results from Two Mediterranean Marine Protected Areas. Mediterr. Mar. Sci..

[30] Giansante C., Fatigati M., Ciarrocchi F., Milillo G.S., Onori L., Ferri N. (2010). Monitoring of Ichthyic Fauna in Artificial Reefs Along the Adriatic Coast of the Abruzzi Region of Italy. Vet. Ital..

[31] Istituto Zooprofilattico Sperimentale (IZS) (2016). Monitoraggio Biologico Sulle Barriere Artificiali Installate in Provincia di Pescara, 10° Anno di Monitoraggio (Anno 2014) e Relazione Finale 2005–2014.

[32] Istituto Zooprofilattico Sperimentale (IZS) (2017). Monitoraggio Biologico Sulle Barriere Artificiali Installate in Prossimità del Comune di Cologna. Reports 2006–2015.

[33] Istituto Zooprofilattico Sperimentale (IZS) (2017). Monitoraggio Biologico Sulle Barriere Artificiali Installate in Prossimità della Torre del Cerrano. Reports 2005–2015.

[34] Istituto Zooprofilattico Sperimentale (IZS) (2017). Monitoraggio Biologico Sulle Barriere Artificiali Installate in Prossimità dei Comuni di Martinsicuro e Alba Adriatica. Reports 2007–2015.

[35] Nieder J., La Mesa G., Vacchi M. (2000). Blenniidae Along the Italian Coasts of the Ligurian and Tyrrhenian Sea: Community Structure and New Records of *Scartella cristata* for Northern Italy. Cybium.

[36] Kagerer M., Patzner R.A. (2017). Depth Distribution and Habitat Utilization of Blennies (Blenniidae & Tripterygiidae) on Two Differently Exposed Coastal Areas on the Island of Lošinj, Croatia. Bull. Fish Biol..

[37] De Francesco M.C., Cerrano C., Pica D., D’onofrio D., Stanisci A. (2017). Characterization of Teatine Coast Marine Habitats (Central Adriatic Sea) Toward an Integrated Coastal Management. Oceanogr. Fish..

[38] Machado Toffolo M., Pongiluppi E., Goffredo S. (2022). Sea Sentinels: Divers United for the Environment. Annu. Rep..

[39] Sushi Drop Project Report on the Scientific Survey Operated with the UUV System in the Second Selected Area. https://programming14-20.italy-croatia.eu/documents/291445/4971848/Act_5.2_D5.2.2_Report_on_the_scientific_survey_operated_with_the_UUV_system_in_the_second_selected_area.pdf/bd639653-e26f-87c9-c711-d29be12f5ece?t=1647848394392.

[40] Cheminée A., Rider M., Lenfant P., Zawadzki A., Mercière A., Crec’Hriou R., Pastor J. (2017). Shallow Rocky Nursery Habitat for Fish: Spatial Variability of Juvenile Fishes Among This Poorly Protected Essential Habitat. Mar. Pollut. Bull..

[41] Cheminée A., Le Direach L., Rouanet E., Astruch P., Goujard A., Blanfuné A., Harmelin-Vivien M. (2021). All Shallow Coastal Habitats Matter as Nurseries for Mediterranean Juvenile Fish. Sci. Rep..

[42] Zorica B., Čikeš Keč V., Vrgoč N., Isajlović I., Piccinetti C., Mandić M., Pešić A. (2020). A Review of Reproduction Biology and Spawning/Nursery Grounds of the Most Important Adriatic Commercial Fish Species in the Last Two Decades. Acta Adriat..

[43] Imbert M. (2014). Snorkel Surveys of the Marine Environment: Methodological Guide. MedPAN Collection.

[44] Nalmpanti M., Chrysafi A., Meeuwig J.J., Tsikliras A.C. (2023). Monitoring Marine Fishes Using Underwater Video Techniques in the Mediterranean Sea. Rev. Fish Biol. Fish..

[45] Harmelin J.-G. (1987). Structure et Variabilité de l’Icthyofaune d’une Zone Rocheuse Protégée en Méditerranée (Parc National de Port-Cros, France). Mar. Ecol..

[46] Prato G., Thiriet P., Di Franco A., Francour P. (2017). Enhancing Fish Underwater Visual Census to Move Forward Assessment of Fish Assemblages: An Application in Three Mediterranean Marine Protected Areas. PLoS ONE.

[47] Jones R.S., Thompson M.J. (1978). Comparison of Florida Reef Fish Assemblages Using a Rapid Visual Technique. Bull. Mar. Sci..

[48] Murphy H.M., Jenkins G.P. (2010). Observational Methods Used in Marine Spatial Monitoring of Fishes and Associated Habitats: A Review. Mar. Freshw. Res..

[49] Syms C. (1995). Multi-Scale Analysis of Habitat Association in a Guild of Blennioid Fishes. Mar. Ecol. Prog. Ser..

[50] De Martini E.E., Roberts D. (1982). An Empirical Test of Biases in the Rapid Visual Technique for Species-Time Censuses of Reef Fish Assemblages. Mar. Biol..

[51] Di Serafino A., Arbuatti A. Evaluation of Coastal Aquatic Animal Biodiversity in the Trabocchi Coast (CH): The Importance of Ecosystem Health and Wildlife Conservation Within the Context of Growing Human Activities. Proceedings of the Conservation Medicine and Wildlife Health International Seminar.

[52] Fuschi M., Cilli A. (2023). Nuovi, Verosimili Modelli di Sviluppo Turistico Locale: Le Prospettive della Costa dei Trabocchi. Geotema.

[53] Fedele L., Nanni A. Coastal Heritage for Adaptive and Inclusive Strategies: The Case of the Trabocchi Coast Along the Mid-Adriatic. Proceedings of the 14th Biennale of European Towns and Town Planners.

[54] Dulčić J., Grbec B., Lipej L. (1999). Information on the Adriatic Ichthyofauna: Effect of Water Warming?. Acta Adriat..

[55] Calafat F.M., Frederikse T., Horsburgh K. (2022). The Sources of Sea-Level Changes in the Mediterranean Sea Since 1960. J. Geophys. Res. Oceans.

[56] Lipej L., Kovačić M., Dulčić J. (2022). An Analysis of Adriatic Ichthyofauna—Ecology, Zoogeography, and Conservation Status. Fishes.

[57] Parras-Berrocal I.M., Vázquez R., Cabos W., Sein D.V., Álvarez O., Bruno M., Izquierdo A. (2023). Dense Water Formation in the Eastern Mediterranean Under a Global Warming Scenario. Ocean Sci..

[58] Castriota L., Falautano M., Perzia P. (2024). When Nature Requires a Resource to Be Used—The Case of *Callinectes sapidus*: Distribution, Aggregation Patterns, and Spatial Structure in Northwest Europe, the Mediterranean Sea, and Adjacent Waters. Biology.

[59] Wang J., Dai J., Gao W., Yao X., Dewancker B.J., Gao J., Zeng J. (2024). Achieving Sustainable Tourism: Analysis of the Impact of Environmental Education on Tourists’ Responsible Behavior. Sustainability.

[60] Esposito E.M., Palumbo D., Lucidi P. (2020). Traveling in a Fragile World: The Value of Ecotourism. Problematic Wildlife II: New Conservation and Management Challenges in the Human-Wildlife Interactions, Angelici, F.M., Rossi, L.R., Eds..

[61] Comitato Capitale Naturale (2022). Quinto Rapporto Sullo Stato del Capitale Naturale in Italia.

[62] Harmelin J.-G. (1999). Visual Assessment of Indicator Fish Species in Mediterranean Marine Protected Areas. Nat. Sicil..

[63] Bombace G., Fabi G., Fiorentini L., Spagnolo A. Assessment of the Ichthyofauna of an Artificial Reef through Visual Census and Trammel Net: Comparison between the Two Sampling Techniques. Proceedings of the 30th European Marine Biological Symposium.

[64] Guidetti P. (2000). Differences among Fish Assemblages Associated with Nearshore *Posidonia oceanica* Seagrass Beds, Rocky-Algal Reefs, and Unvegetated Sand Habitats in the Adriatic Sea. Estuar. Coast. Shelf Sci..

[65] Castellarin C., Visintin G., Odorico R. (2001). Ittiofauna della Riserva Naturale Marina di Miramare (Golfo di Trieste, alto Adriatico). Annales Ser. Hist. Nat..

[66] Fabi G., Grati F., Lucchetti A., Trovarelli L. (2002). Evolution of the Fish Assemblage around a Gas Platform in the Northern Adriatic Sea. ICES J. Mar. Sci..

[67] Cenci E., Mazzoldi C. (2005). Le Tegnue di Chioggia: Un’Analisi Qualitativa e Quantitativa della Fauna Ittica. Riassunti del 36° Congresso Nazionale Della Società Italiana di Biologia Marina.

[68] Fiorin R., Cerasuolo C., Curiel D., Riccato F. (2008). Il Popolamento Ittico e Macroalgale delle Scogliere del Litorale Veneziano: Interazione tra le Alghe Brune del Genere *Cystoseira* e Alcune Specie di Pesci. Biol. Mar. Mediterr..

[69] Riccato F., Fiorin R., Curiel D., Rismondo A., Cerasuolo C., Torricelli P. (2009). Interazione tra il Popolamento Ittico e le Alghe Brune del Genere *Cystoseira* in un Ambiente di Scogliera Artificiale del Golfo di Venezia. Boll. Mus. Civ. Stor. Nat. Venezia.

[70] Fabi G., Grati F., Manoukian E., Spagnolo A. (2011). Sintesi dei Monitoraggi Volti a Valutare gli Impatti di Nuove Piattaforme Offshore nell’Area Interessata alla Realizzazione della Piattaforma Elettra. Tech. Rep..

[71] Guidetti P., Bussotti S., Di Franco A., Di Lorenzo M., Izzi C. (2011). Relazione Finale Monitoraggio delle Specie Ittiche Focali (Tremiti). https://www.parcogargano.it/upload/parcodelgargano/gestionedocumentale/relazione%20finale_784_2126.pdf.

[72] Riccato F., Fiorin R., Penzo P., Da Ros L., Boldrin A. (2011). Ittiofauna Associata ad una Barriera Artificiale in Nord Adriatico. Boll. Mus. Stor. Nat. Venezia.

[73] De Gioia M., Dalle Mura I., D’Onghia F.M., Strippoli G., Costantino G., Barbone E., Ungaro N. (2022). The Role of Scientific Divers in the ADRIREEF Project: ARPA Puglia Activities. Ninth International Symposium “Monitoring of Mediterranean Coastal Areas: Problems and Measurement Techniques”.

