# Supplementary material for: Long-Term Ecosystem Monitoring Along the Trabocchi Coast (Chieti, Italy): Insights from Underwater Visual Surveys (2011–2024)
Source: Animals (Basel). 2024 Dec 1;14(23):3469. doi: 10.3390/ani14233469 (PMC11639999; doi:10.3390/ani14233469)
Supplement: Supplementary file 1 [file animals-14-03469-s001.zip › animals-3311205-supplementary.pdf]

Table S1. Monitoring of marine species in the western Adriatic outside Abruzzo's coastlines*

Site	Duration (years)	Method	Bathymetry	Substrates	n. fish species	other taxa (n.)	Authors
Senigallia (AN)	---	---	---	artificial reef	15	---	[63]
Otranto (BR)	1	UVC diving	-6.5/-8.0 m	*P. oceanica* beds	34	---	[64]
Tremiti (FG)				rocky reefs, bare sand	20		
Miramare (TS)	1	UVC	-0,5/ -5 m	reef	50	---	[65]
				reef e offshore	64		
				Miramare park	101		
Gas platform (RN)	2	nets	---	silt-clayey bottom	49	Molluscae (6)<br>Crustacea (3)	[66]
Chioggia (VE)	2	UVC diving	---	natural reef	32	---	[67]
Venezia (VE)				artificial reef	31		[68]
Pellestrina (VE)	3	UVC diving	-4/-5 m	artificial reef	32	Macroalgae (62)	[69]
Gas platform (AN)	---	nets	---	pelite sediments	22	Molluscae (6)<br>Crustacea (3)	[70]
Tremiti (FG)	2	UVC diving	---	multiple substrates	79	---	[71]
Costa cavallino (VE)	1	UVC diving	-14 m	artificial reef	25	---	[72]
Torre Guaceto (BR)	3	UVC diving, rover	-24/-29 m	biogenic reefs	18	Porifera (23)	[73]

* Results are ordered according to the publication years. Many references are in the form of reports (grey literature).
